# A Snapshot of Potentially Inappropriate Prescriptions upon Pediatric Discharge in Oman

**DOI:** 10.3390/pharmacy10050121

**Published:** 2022-09-23

**Authors:** Alaa M. Soliman, Ibrahim Al-Zakwani, Ibrahim H. Younos, Shireen Al Zadjali, Mohammed Al Za’abi

**Affiliations:** 1 Department of Pharmacology and Clinical Pharmacy, College of Medicine and Health Sciences, Sultan Qaboos University, Muscat PC 123, Oman; 2 Department of Clinical Pharmacology, College of Medicine, Menoufia University, Shebin El Koum 51132, Egypt; 3 Physician Assistant Program, Morsani College of Medicine, University of South Florida, Tampa, FL 33620, USA; 4 Department of Pharmacy, Sultan Qaboos University Hospital, Muscat PC 123, Oman

**Keywords:** inappropriate prescribing, inappropriate medications, potentially prescribing omission, pediatrics

## Abstract

*Background:* Identifying and quantifying potentially inappropriate prescribing (PIP) practices remains a time-consuming and challenging task, particularly among the pediatric population. In recent years, several valuable tools have been developed and validated for assessing PIP. This study aimed to determine the prevalence of PIP and related risk factors in pediatric patients at a tertiary care hospital in Oman. *Materials and Methods:* A retrospective study was conducted by reviewing the medical records of pediatric patients (<18 years) from 1 October to 31 December 2019. Potentially inappropriate medication (PIM) and potential prescribing omission (PPO) were assessed using an internationally validated pediatric omission of prescriptions and inappropriate prescriptions (POPI) tool. *Results:* A total of 685 patients were included; 57.5% were male, and 30.5% had at least one comorbidity. Polypharmacy was identified in 70.2% of these patients, with a median of 2 (1–3) medications. PIM was observed in 20.4% of the cohort, with the highest in ENT-pulmonary disease (30.5%), followed by dermatological disorders (28.6%). PPO was identified in 6.9% of the patients with digestive and neuropsychiatric disorders, with the highest rate of 54% and 24%, respectively. Age (*p* = 0.006), number of medications (*p* = 0.034), and prescriber rank (*p* = 0.006) were identified as significant predictors of PIM, whereas age (*p* = 0.044) was the only significant predictor for PPO. *Conclusions:* The rates of PIM and PPO were high in this study population. In light of these findings, educational and interventional activities and programs are needed.

## 1. Introduction

Prescribing in pediatrics is often complicated in terms of safety and pharmacology because of the lack of sufficient evidence on these parameters within this group and because most prescriptions are based on observational studies obtained from adults [1,2,3]. Therefore, inappropriate prescriptions are a major concern in this population. Several tools, including Beer’s criteria and STOP/START criteria (screening tools for older persons’ prescriptions and screening tools to alert doctors to the right treatment), have been developed to determine potentially inappropriate prescribing (PIP). However, the main targets of these tools are geriatric and adult populations, and the development of specific tools for the detection and screening of PIP in pediatrics has gained popularity just in the last decade [4,5,6]. 

The first reported detection tool for pediatric patients to screen for potentially prescribing omissions (PPO) and potentially inappropriate medications (PIM), both comprising PIP, was developed by Prot-Labarthe et al. [7]. It was a mixed tool containing 105 explicit and implicit items and termed ‘pediatrics omission of prescriptions and inappropriate prescriptions’ (POPI). A modified POPI tool that incorporates a mixture of guidelines and practices of France, the UK, and the US was developed by Corrick et al. [8]. In 2020, a group of international experts from 12 countries reported the development and validation of a POPI tool containing 73 PIM/PPOs in different fields [9].

The use of PIM/PPO tools has enabled researchers to more specifically determine the rate of PIP in the pediatric population. In 2014, Barry et al. [10] conducted a cross-sectional study to assess the overall prevalence of PIP in children in primary care and analyzed PIP prevalence by sex in Ireland. The average prevalence of PIP by commission and omission in eligible children aged < 16 years was 3.5% and 2.5%, respectively. Berthe-Aucejo et al. were the first to estimate the prevalence of PIM and PPO using the POPI tool in pediatric patients within a hospital and community pharmacy [11]. Herein, the PIM and PPO rates were 3.3% and 2.7% at the hospital and 26.4% and 11.3% at the community pharmacy, respectively, with the highest rate of PIM observed among those with respiratory and digestive diseases. 

In Oman, there are no documented reports of PIP in pediatric patients. We were able to retrieve two retrospective cross-sectional studies that were conducted among the pediatric population in Oman, but these only described the common drugs and drug classes used at the respective centers [12,13]. A survey conducted by Al-Maqbali et al. in a polyclinic in 2018 suggests that 83.3% of drugs prescribed for children were from the World Health Organization (WHO)-approved drug list, indicating that the prescription process was rational [14]. However, there is no information on PIM and PPO. Therefore, this study aimed to assess the prevalence of PIM and PPO in the discharge prescriptions of pediatric patients admitted to a tertiary care center in Oman using the internationally validated POPI tool.

## 2. Material and Methods

### 2.1. Setting and Design

This retrospective, descriptive study was conducted at Sultan Qaboos University Hospital (SQUH) in 2019. SQUH is an academic tertiary care hospital with almost 500 beds located in Muscat, Oman. In 2021, SQUH had 350,498 patients’ visits, of which 7.1% (*n* = 25,630) were from pediatric wards (72 beds) and clinics. 

### 2.2. Inclusion and Exclusion Criteria

The inclusion criteria were patients aged < 18 years with a prescription with at least one medication for any condition/s mentioned in the POPI tool. Patients with other conditions or no medication at discharge were excluded. 

### 2.3. Data Collection

Data were collected from the electronic patient records (EPR), a system used by SQUH for storing patient information such as demographics, diagnosis, number of drugs per prescription, comorbidities, and prescribers’ ranks. The prescription or consumption of two or more different medications for at least one day was defined as polypharmacy, as previously described by Bakaki et al. [15].

### 2.4. POPI Tool

The internationally validated POPI tool described by Berthe-Aucejo et al. was used to determine PIM and PPOs [11]. The information on the last episode in the patient’s discharge prescription was manually evaluated to determine PIM/PPO. The prevalence of PIM was defined as “the percentage of patients with at least one PIM,” and the prevalence of PPO as “the percentage of patients with at least one PPO.” A randomly selected 25% of the PIM and PPO events were examined and verified by the co-authors’ MA (MD) (and SA (clinical pharmacist). 

### 2.5. Sample Size Estimation and Statistical Analysis

Based on the results of previous studies, the prevalence of PIM and PPO at SQUH was estimated at approximately 11% [11]. The minimum effective sample size was 410 patients for a 95% confidence level with a 3% estimated margin of error. To compensate for missing data and cover a minimum period of 3 months (quartile of a year), the sample size was further adjusted to 685 patients.

Descriptive statistics were used to describe the data. Continuous data with non-normally distributed variables were described as medians and interquartile ranges. Categorical data were expressed as frequencies and percentages. Pearson’s chi-squared test was employed to estimate the association between PIM/PPO and other categorical characteristics. Furthermore, the Mann–Whitney test was used to assess the significance of differences between the ranks of the two groups of non-normally distributed continuous variables. To determine the impact of various independent risk factors on PIMs (yes/no) and PPOs (yes/no), multiple logistic regression models were employed using a simultaneous method. Adjusted odds ratios (ORs) were calculated, along with their 95% confidence intervals. The goodness of fit of the multiple logistic models was examined using Hosmer and Lemeshow goodness-of-fit statistics with P values of >0.05, denoting good model fit [16]. Statistical significance was set at *p* < 0.05. Data were analyzed using Statistical Package for Social Sciences software version 25 (SPSS).

## 3. Results

### 3.1. Demographic and Clinical Data

Table 1 shows the demographic and clinical characteristics of the study participants (*N* = 685). Gender distribution showed that more than half of the patients were male (57.5%). The median age was 5.8 (2.2–10.8) years, with the majority of patients in the age group of 6–12 years (30.1%) followed by 2–6 years (28.5%), 1 month–2 years (22.2%), and 12–18 years (19.3%). Most prescriptions were for outpatients (85.8%). 

Only 30.5% of the pediatric patients had comorbidities. The majority (24.8%) of the patients had only one comorbidity. The most common comorbidities were allergic rhinitis, asthma, and global developmental delay, at 8.3%, 4.2%, and 2.6%, respectively. 

The median number of prescribed medications was two (1–3). Overall, 70.2% of patients presented with polypharmacy. The percentages of patients prescribed one, two, and three or more medications were 29.8%, 31.8%, and 38.4%, respectively. Specialists prescribed medications for 35.2% of the subjects, followed by residents (31.2%) and consultants (21%).

ENT pulmonary disorders were present in 419 prescriptions (42.4%), followed by various illnesses (264, 26.7%), neuropsychiatric (137, 13.9%), digestive (85, 8.6%), and dermatological (83, 8.4%) disorders.

### 3.2. PIMs and PPOs

Among the studied patients, 140 (20.4%) presented with at least one PIM and 47 (6.9%) with at least one PPO. About 18.4% had one PIM (*n* = 126) and nearly 2% (*n* = 14) had two PIMs. A total of 6.4% (*n* = 44) of the subjects had only one PPO and almost 0.4% (*n* = 3) had two PPOs.

Table 2 and Table 3 present the prevalence of PIM and PPO in patients according to the system/disorder and criteria, respectively. The most prevalent PIM with ENT-pulmonary disorders included H_l_-antagonists with sedative or atropine-like effects before 30 months of age (25.5%, 12/47), while the most prevalent PPO was omitting 0.9% NaCl to relieve nasal congestion in patients with bronchiolitis (40%, 4/10), and combining paracetamol with antibiotic treatment to relieve pain in ear infections (40%, 4/10).

Prescribing a medication other than paracetamol as a first-line treatment was the most prevalent PIM for several illnesses (54.8%, 17/31), and failure to administer an osmotic laxative to patients treated with morphine for more than 48 h was the only recorded PPO in this category.

Prescribing more than one application of dermocorticoid per day in patients with eczema was the most frequently recorded PIM for dermatological disorders (52.3%, 23/44), and no PPO was recorded in this category. 

The prescription of domperidone to treat vomiting (68.2%, 15/22) and desmopressin nasal spray to treat nocturnal enuresis (60%, 6/10) were the most common PIM for digestive and neuropsychiatric disorders, respectively. The most common PPO in these two disorders was omitting oral rehydration solution in the event of vomiting (70.4%, 19/27) and not recording the growth chart parameters (height and weight) following methylphenidate (100%, 12/12) prescription.

### 3.3. Associated Variables and Risk Factors for PIM and PPO

Table 4 shows the associations of various variables with PIM and PPO. Age (*p* = 0.006), polypharmacy (*p* = 0.034), and prescriber rank (*p* = 0.001) were associated with PIM. Multiple logistic regression analysis showed that PIM was likely in children of ages 2–6 years (odds ratio (OR), 0.47; 95% confidence interval (CI): 0.27–0.82; *p* = 0.008) and those aged 6–12 years (OR, 0.43; 95% CI:0.24–0.76; *p* = 0.004). Furthermore, interns/medical officers were also less likely to be associated with PIM than consultants and above (OR, 0.16; 95% CI: 0.05–0.49; *p* = 0.001). However, the higher the number of discharge medications, the more likely the children to be associated with PIM (OR, 1.26; 95% CI:1.02–1.56; *p* = 0.034). The only significant predictor associated with PPO was age, with children 6–12 years less likely to be associated with PPO compared with those <2 years (OR, 0.39; 95% CI:0.16–0.98; *p* = 0.044). 

## 4. Discussion

PIP detection tools are useful prognostic tools for adverse drug events (ADEs), emergency department (ED) visits, and hospitalizations. Wallace et al. [17] show that 74% of those identified as having PIP at the baseline reported one or more ADE at follow-up, while Cahir et al. [18] report that patients with two or more PIPs were twice as likely to experience ADEs and ED visits.

Several studies using PIP detection tools have focused on the detection of PIM and PPO in geriatric populations [19,20]. In the pediatric population, the POPI tool has an advantage over other tools (e.g., STOPP/START, Beer’s criteria) because it is developed to address diseases that primarily affect pediatrics. The spectrum of diseases affecting children differs from those affecting geriatrics. For example, psychiatry and cardiology are the main categories in most tools used in geriatrics, whereas respiratory, gastroenterology, and dermatology disorders are the main items in the POPI tool. As few studies and centers utilized this tool, its disadvantage is not yet evident [9,11].

We screened 685 discharge prescriptions of inpatients and outpatients aged < 18 years at SQUH to identify PIM and PPO using the internationally validated POPI tool. Thus, to the best of our knowledge, this is the first study to observe the prevalence of PIM and PPO in a pediatric population, not only in Oman but also in the Middle East region on the whole.

The findings revealed that 20.4% of the patients had PIM, of which 18.4% had only one PIM, and nearly 2% had two PIMs. Most observed PIMs were associated with ENT pulmonary disorders (30.5%), followed by dermatological conditions (28.6%). In comparison to a previous study conducted in France by Berthe-Aucejo et al. [11]. the prevalence of PIM among patients discharged from the ED was 3.3%. In contrast, it was 26.4% among patients from community pharmacies. ENT pulmonary and digestive disorders showed the highest rates of PIM in hospital and community pharmacies.

In patients with ENT infections, prescription of H_l_-antagonists with sedative or atropine-like effects (chlorpheniramine) before 30 months of age represented 25.5% of the total PIMs for ENT-pulmonary disorders, notably higher than that reported previously (0.8%) [11]. Prescribing azithromycin or clarithromycin as a first-line antibiotic instead of amoxicillin for acute otitis media and strep sinusitis was observed in 19.1% of ENT-pulmonary disorders compared with 12.5% in the French study [11]. Azithromycin and clarithromycin, as compared with amoxicillin, were prescribed once instead of thrice daily for a shorter duration. Thus, adherence may be more convenient and easier for parents and patients, which can explain such a trend to an extent.

Prescribing nasal or oral decongestants, such as Clarinase^®^ or Actifed^®^, represented 14.9% of the total PIM in ENT-pulmonary disorders, which was significantly higher than that reported previously (0.2%) [11]. Dermatological disorders were ranked second after ENT pulmonary disorders in our study. Prescribing of topical corticosteroid application more than once per day was observed in 52.3% of the total PIM in this category. Once-daily application of corticosteroids is effective and convenient, with a lower risk of side effects and costs [21].

Prescribing a medication other than acetaminophen/paracetamol, such as non-steroidal anti-inflammatory drugs (NSAIDs) as a first-line treatment, represented 54.8% of the total PIM in various illnesses, followed by rectal administration of paracetamol (25.8%). Compared with paracetamol, NSAIDs are associated with more severe adverse events in children [22]. Domperidone represented 68.2% of the total PIM in digestive disorders, although it causes cardiac side effects such as QT prolongation in the adult and pediatric populations [23].

Prescribing desmopressin as a nasal spray for the treatment of nocturnal enuresis accounted for 60% of all PIMs in neuropsychiatric disorders. Nasal desmopressin is associated with a higher incidence of symptomatic hyponatremia than oral desmopressin and is no longer the preferred route of administration [24].

We identified that 6.9% of the patients had PPO; among them, 6.4% had only one omitted medication, while 0.4% had two PPOs. The French study revealed that 2.7% and 11.3% of patients had omitted medications at the hospital and community pharmacies, respectively [11]. The most common PPO was within digestive disorders, with a prevalence of 54%, of which 70.4% and 29.6% were due to the omission of oral rehydration solution in the event of vomiting and diarrhea, respectively. Complications due to unattended dehydration caused by diarrhea and vomiting include electrolyte imbalance, fainting, and heart rhythm abnormalities [25]. The second most common PPO was not recording a growth chart (height and weight) in patients with neuropsychiatric disorders (24%) prescribed methylphenidate. Long-term methylphenidate use in children has been linked to decreased weight gain and growth retardation [26].

Respiratory disorders accounted for 20% of the total PPOs. This was due to the omission of paracetamol in the treatment of ear infections to relieve pain (40%); the use of 0.9% NaCl to relieve nasal congestion (40%), and the use of inhaled corticosteroids as a preventative treatment for persistent asthma (20%). In the French study, the prevalence of omitting paracetamol for ear infections was 25.5% lower than that in our study, whereas omitting 0.9% NaCl for nasal congestion was 74.5%, markedly higher than that observed in our study [11].

Screening for risk or associated factors with PIM and PPO showed a significant association between the prevalence of PIM and prescriber rank (*p* = 0.001), with interns/medical officers less likely to be associated with PIM more than consultants and above. These findings contradict those reported by Ryan et al. [27]. In the current study, the number of prescriptions ordered by interns/medical officers was minimal (12.6%) compared with other ranks. It is also possible that interns/medical officers are less likely to write prescriptions with PIMs or PPOs, as their prescriptions require further verification and approval by a specialist or a consultant. Seden et al. [28] conclude that prescriber experience in primary care does not impact the overall error rate. Multiple logistic regression analysis did not reveal any association between PPOs and prescription rank (*p* = 0.486). PIM was also likely to be associated with polypharmacy (85% vs. 15%; *p* < 0.001). Polypharmacy is a strong predictor of drug reactions in both adults and children [29,30]. In this study, age was a significant independent predictor of PIM for the age groups–2–6 years and PIM and PPO in the 6–12 years age group. The reason for this finding is unknown and requires further investigation. 

Our study has some limitations. First, since this is a monocentric design, the findings may not be generalizable to other centers. Second, due to the retrospective nature of the study, we could only predict the associative relationship but not the causal link. Third, data from the EPR were the only source of information; hence, the judgment was based solely on this data, without other verification or additional references. As a result, it is possible that some clinically based or real-time judgments were made but not registered in the electronic system. Fourth, because our center is a tertiary care facility, some conditions were absent; therefore, some of the POPI criteria could not be studied.

## 5. Conclusions

In summary, the estimated prevalence of PIM and PPO using the POPI tool at SQUH might represent a snapshot of PIM/PPO in the Omani pediatric population. The results suggested a need for a more comprehensive assessment of patients’ prescriptions by pharmacists to optimize prescriptions and minimize PIP before their discharge. In addition, a study with a larger sample size at different hospital settings, that is, primary, secondary, and tertiary care centers, is warranted for better statistical inferences and generalizability of the findings. Furthermore, future research on the clinical impact of the identified PIMs and PPOs is warranted.

## Figures and Tables

**Table 1:** Demographic and clinical characteristics of studied population (*N* = 685).

Variable		*N* (%)
Gender	Male	394 (57.5)
	Female	291 (42.5)
Age group	1 month–2 years	152 (22.2)
	2–6 years	195 (28.5)
	6–12 years	206 (30.1)
	12–18 years	132 (19.3)
Prescription type	Inpatient	97 (14.2)
	Outpatient	588 (85.8)
Co-morbidities (*n* = 209)	1	170 (29.8)
	2	29 (4.2)
	3	6 (0.9)
	5	4 (0.6)
Number of medications per prescription	1	204 (29.8)
	2	218 (31.8)
	≥3	263 (38.4)
Prescriber’s rank	≥Consultant	144 (21)
	Specialist	241 (35.2)
	Resident	214 (31.2)
	Intern & medical officer	86 (12.6)
Number of disorders in all prescriptions (*n* = 988)	Digestive	85 (8.6)
	ENT-pulmonary	419 (42.4)
	Dermatological	83 (8.4)
	Neuropsychiatric	137 (13.9)
	Various illnesses	264 (26.7)

ENT: ear, nose, and throat.

**Table 2:** PIMs frequency and percentage in relation to disorders identified by POPI tool (*N* = 154).

Criteria/Disorders		Number and Proportion of PIMs in Relation to Total PIMs (%)	PIMs in Relation to Total PIMs in the Disorder (%)
Various illnesses		31/154 (20.1%)	
	AI-1 Prescription of two alternating antipyretics as a first-line treatment	6/154 (3.9%)	6/31 (19.4%)
	AI-2 Prescription of a medication other than paracetamol as a first line treatment (except in the case of migraine)	17/154 (11%)	17/31 (54.8%)
	AI-3 Rectal administration of paracetamol as a first-line treatment	8/154 (5.2%)	8/31 (25.8%)
Digestive disorders		22/154 (14.3%)	
	EI-1 Metoclopramide	3/154 (1.9%)	3/22 (13.6%)
	EI-2 Domperidone	15/154 (9.7%)	15/22 (68.2%)
	EI-3 Gastric antisecretory drugs to treat gastroesophageal reflux, dyspepsia, the crying of new-born babies (in the absence of any other signs or symptoms), as well as faintness in infants	4/154 (2.6%)	4/22 (18.2%)
ENT-Pulmonary disorders		47/154 (30.5%)	
	GI-1 Mucolytic drugs, mucokinetic drugs, or helicidine before two years of age	8/154 (5.2%)	8/47 (17.0%)
	HI-1 Beta2 agonists, corticosteroids to treat an infant’s first case of bronchiolitis	2/154 (1.3%)	2/47 (4.3%)
	II-1 An antibiotic other than amoxicillin as a first-line treatment for acute otitis media, strep throat, or sinusitis (provided that the patient is not allergic to amoxicillin).	9/154 (5.8%)	9/47 (19.1%)
	II-4 Antibiotics to treat otitis media with effusion (OME), except in the case of hearing loss or if OME lasts for more than three months	2/154 (1.3%)	2/47 (4.3%)
	II-5 Corticosteroids to treat acute suppurative otitis media, nasopharyngitis, or strep throat	3/154 (1.9%)	3/47 (6.4%)
	II-6 Nasal or oral decongestant (oxymetazoline, pseudoephedrine, naphazoline, ephedrine, tuaminoheptane, phenylephrine)	7/154 (4.5%)	7/47 (14.9%)
	II-7 Hl-antagonists with sedative or atropine-like effects (pheniramine, chlorpheniramine), or camphor; inhalers, nasal sprays, or suppositories containing menthol (or any terpene derivatives) before 30 months of age	12/154 (7.8%)	12/47 (25.5%)
	II-8 Ear drops in the case of acute otitis media	4/154 (2.6%)	4/47 (8.5%)
Dermatological disorders		44/154 (28.6%)	
	KI-2 The combined use of an oral and a local antibiotic	4/154 (2.6%)	4/44 (9.1%)
	KI-3 Oral or local antibiotics as a monotherapy (not in combination with another drug)	3/154 (1.9%)	3/44 (6.8%)
	NI-1 The combination of locally applied and orally administered antibiotic	1/154 (0.6%)	1/44 (2.3%)
	PI-1 A strong dermocorticoid (clobetasol propionate 0.05% Dermoval, betamethasone dipropionate Diprosone) applied to the face, the armpits or groin, and the backside of babies or young children	1/154 (0.6%)	1/44 (2.3%)
	PI-2 More than one application per day of a dermocorticoid, except in cases of severe lichenification	23/154 (14.9%)	23/44 (52.3%)
	PI-3 Local or systemic antihistamine during the treatment of outbreaks	8/154 (5.2%)	8/44 (18.2%)
	PI-4 Topically applied 0.03% tacrolimus before two years of age	1/154 (0.6%)	1/44 (2.3%)
	PI-5 Topically applied 0.1% tacrolimus before 16 years of age	2/154 (1.3%)	2/44 (4.5%)
	PI-6 Oral corticosteroids to treat outbreaks	1/154 (0.6%)	1/44 (2.3%)
Neuropsychiatric disorders		10/154 (6.5%)	
	SI-1 Desmopressin administered by a nasal spray.	6/154 (3.9%)	6/10 (60%)
	SI-2 Desmopressin in the case of daytime symptoms	3/154 (1.9%)	3/10 (30%)
	UI-3 Slow-release methylphenidate as two doses per day, rather than only one dose	1/154 (0.6%)	1/10 (10%)

PIMs: potentially inappropriate prescribing medications, POPI: paediatrics omission of prescriptions and inappropriate prescriptions, ENT: ear nose and throat.

**Table 3:** PPOs frequency and percentage in relation to disorders identified by POPI tool (*N* = 50).

Criteria/Disorders		Number and Proportion of PPOs in Relation to Total PPOs (%)	PPOs in Relation to Total PPOs in the Disorder (%)
Various illnesses		1/50 (2%)	
	AO-2 Failure to give an osmotic laxative to patients being treated with morphine for a period of more than 48 h	1/50 (2%)	1/1 (100%)
Digestive disorders		27/50 (54%)	
	EO-1 Oral rehydration solution in the event of vomiting	19/50 (38%)	19/27 (70.4%)
	FO-1 Oral rehydration solution in the event of diarrhea	8/50 (16%)	8/27 (29.6%)
ENT-Pulmonary disorders		10/50 (20%)	
	HO-1 0.9% NaCl to relieve nasal congestion (not applicable if nasal congestion is already being treated with 3% NaCl delivered by a nebulizer)	4/50 (8%)	4/10 (40%)
	IO-1 Paracetamol combined with antibiotic treatment for ear infections to relieve pain	4/50 (8%)	4/10 (40%)
	JO-2 Preventative treatment (inhaled corticosteroids) in the case of persistent asthma	2/50 (4%)	2/10 (20%)
Neuropsychiatric disorders		12/50 (24%)	
	UO-1 Recording a growth chart (height and weight) if the patient is taking methylphenidate	12/50 (24%)	12/12 (100%)

PPO: potentially prescribing omissions, POPI: paediatrics omission of prescriptions and inappropriate prescriptions, ENT: ear nose and throat.

**Table 4:** Multiple logistic regression of risk factors for potentially inappropriate medications (PIMs) and potentially prescribing omissions (PPOs).

Variable		PIMs		PPOs	
		Odd Ratio [95% CI]	*p*-Value	Odd Ratio [95% CI]	*p*-Value
Gender		1.01 [0.68–1.49]	0.954	0.79 [0.42–1.47]	0.450
Age group			0.006 *		0.240
	1 month-2 years	1		1	
	2–6 years	0.47 [0.27–0.82]	0.008 *	0.73 [0.33–1.62]	0.439
	6–12 years	0.43 [0.24–0.76]	0.004 *	0.39 [0.16–0.98]	0.044 *
	12–18 years	0.80 [0.44–1.48]	0.476	0.80 [0.32–2.01]	0.629
Comorbidity		0.67 [0.43–1.07]	0.091	0.87 [0.43–1.74]	0.690
Number of discharge medications		1.26 [1.02–1.56]	0.034 *	0.89 [0.58–1.37]	0.587
Prescriber rank			0.006 *		0.486
	>Consultant	1		1	
	Specialist	1.06 [0.62–1.83]	0.830	1.43 [0.51–4.00]	0.493
	Resident	0.97 [0.55–1.73]	0.927	1.86 [0.67–5.18]	0.235
	Intern & medical officer	0.16 [0.05–0.49]	0.001 *	2.34 [0.74–7.46]	0.150

CI: confidence interval, * denotes significance, Hosmer & Lemeshow *p*-values, of the PIMs and PPOs models, were 0.357 and 0.436, respectively, denoting good model fit.

## Data Availability

Not applicable.
